# Suicidal attempts and non-suicidal self-injury during gender affirming hormone therapy (GAHT) – a case report

**DOI:** 10.1192/j.eurpsy.2022.2084

**Published:** 2022-09-01

**Authors:** D. Puzio, K. Palka-Szafraniec, I. Makowska

**Affiliations:** Medical University of Lodz, Child And Adolescent Psychiatry Unit, Lodz, Poland

**Keywords:** suicidal attempt, NSSI, Depression, transsexualism

## Abstract

**Introduction:**

Transsexual adolescents frequently present psychiatric comorbidities and psychopathology among which self-injurious behaviours are prominent (Modrego Pardo et al., 2021). GAHT seems to decrease the rate of mental health problems especially in terms of anxiety, depression and hostility (Heylens et al., 2014). However the impact of exogenous cross-sex hormones on non-suicidal and suicidal self-injurious behaviours is not thoroughly understood (Claes et al., 2015).

**Objectives:**

We present the case of a transsexual boy who was first diagnosed with depression and benign self-injurious behaviours and subsequently - transsexualism.

**Methods:**

He was prescribed a treatment with testosterone depot injections in fortnight intervals. The initiation of testosterone injections co-occurred with the switching of antidepressant drug. Self-injurious behaviours, substance abuse and suicidal attempts were observed regularly after GAHT onset – 10-14 days after a testosterone injection. The lethality and intensity of self-harm was greater than that observed before GAHT. After admission to the psychiatry unit pharmacotherapy was adjusted accordingly to presented symptoms. Remission of self-injurious behaviours followed.

**Results:**

The incidence of self-injury 10-14 days after the injection of depot testosterone overlaps the peak of serum testosterone levels in treated patients (O’Connor et al., 2004). Moreover, a relative serotonin deficiency in a depressed patient may be insufficient to counteract testosterone believed to be driving aggressive tendencies (Batrinos, 2012).

**Conclusions:**

Since psychiatric comorbidity and psychopharmacotherapy in transsexual young population is the rule rather than the exception, careful monitoring and therapy adjustment is crucial for maintaining safety and obtaining best results (Kaltiala et al., 2020).

**Disclosure:**

No significant relationships.

